# Laparoscopic lavage in a purulent peritonitis model: impact on inflammatory proteins

**DOI:** 10.1186/s40001-025-02445-2

**Published:** 2025-03-18

**Authors:** Erik Sinclair, Maria K. Magnusson, Eva Angenete, Mattias Prytz, Viktor Tasselius, Lena Öhman, Eva Haglind

**Affiliations:** 1 Department of Surgery, Scandinavian Surgical Outcomes Research Group, Institute of Clinical Sciences, Sahlgrenska Academy, University of Gothenburg, Gothenburg, Sweden; 2 Department of Surgery, Region Västra Götaland, NU-Hospital Group, Trollhättan, Sweden; 3 Department of Research and Development, NU-Hospital Group, Trollhättan, Sweden; 4 Department of Microbiology and Immunology, Institute of Biomedicine, Sahlgrenska Academy, University of Gothenburg, Gothenburg, Sweden; 5 School of Public Health and Community Medicine, Institute of Medicine, University of Gothenburg, Gothenburg, Sweden; 6 Department of Surgery, Region Västra Götaland, Sahlgrenska University Hospital, Gothenburg, Sweden

**Keywords:** CLP, Hinchey grade III diverticulitis, Peritonitis, Experimental, Laparoscopic lavage, Inflammation, Model

## Abstract

**Background:**

Laparoscopic lavage is an effective, safe, and feasible treatment in patients with perforated diverticulitis with purulent peritonitis. Laparoscopic lavage was introduced without any detailed knowledge regarding the mechanisms of action. The aim of this study was to validate the reproducibility of an animal model of purulent peritonitis and to study the effect of laparoscopic lavage on inflammatory proteins in this model.

**Methods:**

Forty rats, divided into eight groups (*n* = 5) were operated. Six groups underwent cecal ligation and puncture (CLP) causing peritonitis and two groups underwent sham surgery. Three CLP and one sham group received laparoscopic lavage, while the remaining groups acted as time-matched controls. Samples of abdominal fluid and blood were collected after 1, 2 or 3 h and analyzed regarding 92 inflammatory proteins using Olink Target 96 Mouse exploratory panel.

**Results:**

Animals with peritonitis had higher levels of inflammatory proteins such as CCL3, IL17A and IL6 in abdominal fluid and serum compared to sham. The groups treated with laparoscopic lavage had lower levels of inflammatory proteins in both abdominal fluid and serum compared with untreated peritonitis groups, results were most distinct sampled after one hour.

**Conclusion:**

Our animal model is reproducible, and mimics perforated diverticulitis with purulent peritonitis with increased levels of inflammatory proteins in abdominal fluid and serum. The levels of several inflammatory proteins were lower following laparoscopic lavage treatment perhaps indicating the physiological effect of laparoscopic lavage. This model can be used to further explore the mechanisms involved in peritonitis and laparoscopic lavage treatment.

**Supplementary Information:**

The online version contains supplementary material available at 10.1186/s40001-025-02445-2.

## Background

A colonic diverticulum is a sac-like herniation of mucosa and submucosa through the muscular layer of the colonic wall, covered by serosa [1]. The presence of colonic diverticula is called diverticulosis, and it is suggested to be the result of genetic predisposition, lifestyle, and environmental factors, including the microbiome [2]. Diverticulosis is common in Western countries and the prevalence increases with age, with numbers about 50% in populations above 65 years of age [3].

Between 5–25% of patients with diverticulosis develop diverticulitis which in most cases does not require any specific treatment [4], but when the inflammation causes a perforation of the bowel the condition becomes serious and potentially life threatening. Perforated diverticulitis may cause purulent peritonitis requiring emergency surgery, traditionally colon resection with or without stoma formation. In recent years randomized clinical trials have shown that laparoscopic lavage is a feasible and safe treatment alternative to resection surgery in patients with purulent peritonitis [5–10]. Laparoscopic lavage is a relatively simple treatment where, under general anesthesia, the abdomen is insufflated with carbon dioxide to a pressure of 12–15 mmHg and the abdominal cavity is rinsed with large amounts of saline at body temperature until return of clear fluid. Today laparoscopic lavage is used in routine care in patients with purulent peritonitis caused by perforated diverticulitis.

Previous studies have suggested that around 60–70% of patients operated with resection surgery without anastomosis and 30–40% of patients who underwent laparoscopic lavage due to perforated diverticulitis with purulent peritonitis needed further surgery [8–11]. A recent retrospective, national cohort study demonstrated no significant difference in reoperation rates between the treatment alternatives within 90 days [12]. Two years after the index surgery there was a significant difference in need for further surgery, in favor of laparoscopic lavage [13].

During peritonitis, activation of pathogen recognition receptors results in the release of an array of proinflammatory mediators from peritoneal macrophages and mesothelial cells [14]. These mediators include, but are not limited to, tumor necrosis factor (TNF)-α, interleukin (IL)−6, IL-1β and the monocyte chemoattractant protein-1 (MCP-1/CCL2). The effects of these factors include vasodilatation and upregulation of adhesion molecules which result in extravasation and activation of leukocytes [15].

Little is known about the pathophysiological mechanisms behind the laparoscopic lavage treatment and the clinical introduction of the treatment was not preceded by any studies in animal models. To obtain a better understanding of the pathophysiology of laparoscopic lavage studies of experimental models can allow for increased knowledge and improvement of the treatment procedures as well as implementation in other inflammatory intra-abdominal conditions.

## Methods

The aim of this study was to determine the abdominal and systemic inflammatory response in rats with experimental purulent peritonitis induced by cecal ligation and puncture (CLP) and to evaluate how laparoscopic lavage effected the levels of inflammatory proteins in abdominal fluid and serum.

### Animals

Sprague–Dawley rats from Charles-River Laboratory, Calco, Italy, male and female, aged 8–10 weeks, weighing approximately 250–375 g were used. Both sexes were included to enhance the study’s translational relevance, as biological sex can influence drug metabolism, hormonal regulation, and treatment responses. This approach was considered important for obtaining clinically relevant insights. Upon arrival, the animals were housed in the animal facility for at least six days and were monitored daily for any signs of illness.

### Groups

**Fig. 1:**
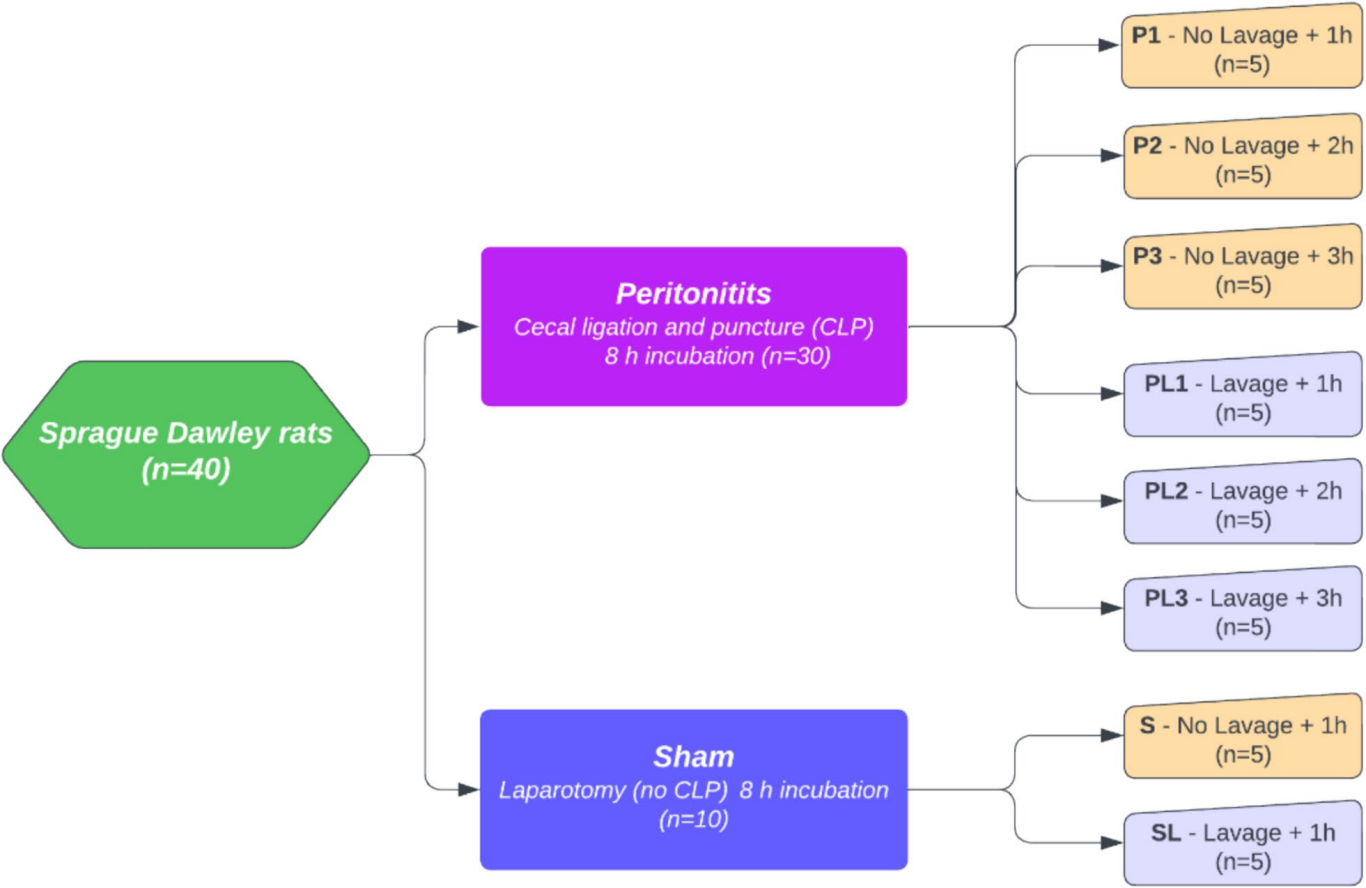
Flowchart for the groups. Forty rats, stratified by sex, were randomized into eight groups (*n* = 5 per group). Six groups were operated with cecal ligation and puncture (CLP) and incubated for 8 h to develop peritonitis. Three groups of peritonitis-afflicted animals were treated with laparoscopic lavage and samples were taken after 1, 2 or 3 h. Groups were named by peritonitis and sampling time in hours (P1, P2, P3) and peritonitis with laparoscopic lavage and sampling time in hours (PL1, PL2, PL3). Two groups were sham operated by laparotomy and incubated for 8 h to match the peritonitis groups. Groups were named by sham (S) and sham treated with laparoscopic lavage (SL) and were sampled after one hour

Forty rats, 20 males/20 females, were randomized into eight groups (*n* = 5), as illustrated in the flowchart (Fig. 1). Randomization was performed by an independent assistant stratifying for sex. The randomization numbers were placed in opaque envelopes and at the start of each operation the surgeon picked a group for each animal by opening an envelope.

### General procedure details

The animal received food and water ad libitum. Temgesic^®^ (buprenorphine) 0,05 mg/kg was given subcutaneously to the animal 30 min prior to induction of anesthesia, and again 6 h later. The animal was placed in an anesthetic box and anesthesia was induced using isoflurane 5% in air 4 L/min. When anaesthetized the animal was moved to the operating table and given a maintenance dose of isoflurane 2.5% in air 3 L/min on a nose cone.

### Cecal ligation and puncture

Purulent peritonitis was induced through the previously described model of cecal ligation and puncture (CLP) followed by 8 h of incubation [16–19]. In short, the anaesthetized animal was put on the operating table so that the abdomen was fully exposed. Through a midline incision the cecum was exteriorized and ligated one centimeter from the cecal apex, using a 4–0 braided ligature. The ligated part of the cecum was punctured straight through using a 1.2 mm needle, creating two holes. A light pressure was applied on the ligated and punctured cecum until a droplet of bowel content was visible at both puncture sites. The cecum was then returned to the abdominal cavity and the abdominal wall was closed in two layers with running sutures using a 4–0 braided suture. The administration of anesthetic gas was terminated, and the animal was allowed to wake up. After 8 h an experimental peritonitis mimicking purulent peritonitis was considered established [16].

### Sham operation

The sham operation was performed as described for CLP but after midline incision and laparotomy the abdominal wall was closed, and the animal was allowed to wake up.

### Lavage

Eight hours after the initial operation the animal was re-anaesthetized and put on the operating table as described above. Close to the midline in the upper left quadrant, the abdominal wall was perforated by a 1.3 × 32 mm (18G), catheter (BD Venflon Pro Safety) to which a 20L electronic endoflator (Karl Storz Endoskop Sverige AB) was connected. Gas pressure was set to 8 mm Hg, using carbon dioxide. Two additional catheters (1.3 × 32 mm) were inserted into the lower left and lower right quadrants of the abdominal cavity, respectively. The animal was tilted slightly to the right with head slightly raised. Saline at room temperature was injected through the catheter in the left lower quadrant in portions of 10 ml, and then passively or by applying suction, drained through the catheter in the right lower quadrant. Between 20–30 ml of saline per animal was used, until return of clear fluid. The total operating time for the lavage procedure from start of anesthesia till return of clear lavage fluid was 8–20 min. All procedures were performed by the same surgeon. The lavage treatment in this study, administered once per animal in the lavage-treated groups, closely replicates the clinical approach used in human diverticulitis treatment.

### Sampling

The animal was re-anaesthetized, and the midline incision was re-opened. A sample of abdominal fluid was taken using an auto pipette. The vena cava was punctured, and a blood sample was collected using a 0.6 × 25 mm (23G) needle and a 3 ml syringe. The animals were euthanized while anesthetized.

The abdominal fluid and blood samples were kept at + 4 °C overnight. The following day the abdominal fluid samples were centrifuged at 4 °C for 6 min at 600G and the blood samples were centrifuged at 4 °C for 7 min at 2000G. The low viscosity part (free from cells) of the centrifuged abdominal fluid and serum from blood samples, were collected and stored at − 80 °C.

The peritonitis-afflicted animals treated with laparoscopic lavage (groups PL1, PL2 and PL3) were sampled 1, 2 or 3 h after treatment, respectively, and the untreated peritonitis animals (groups P1, P2 and P3) were sampled at the corresponding times. The Sham operated animals, S and SL were sampled as stated above, corresponding to “1 h after treatment”.

### Protein analysis

The samples were analyzed regarding 92 proteins by the Proximity Extension Immunoassay (PEA, Olink Proteomics, Uppsala, Sweden) using the Mouse Exploratory panel. In brief, the methodology involves the pair-wise binding of oligonucleotide-labeled antibodies to target proteins, resulting in the generation of a reporter sequence through DNA polymerization. The reporter sequence is amplified, and subsequently detected and quantified using real-time polymerase chain reaction (PCR). Data obtained are normalized to log2 values representing the protein levels in the sample, termed normalized protein expression (NPX), allowing for relative quantification. Absolute comparisons between different proteins cannot be made due to the relative nature of the NPX values. Samples deviating more than 0.3 NPX from the median value of an internal control are considered to fail quality assessment. In this study all samples passed the quality assessment. Proteins that did not meet the minimum level of detection were reported as missing data. Proteins with missing data frequency of  > 40% were excluded from further analyses.

### Statistical analyses

Principal component analyses were conducted on logarithm transformed and scaled data using the prcomp-algorithm and visualized using the pca3d-package in R (version 4.2.2) [20].

Student’s *t*-test was used to compare pair-wise differences between groups for each protein, both for abdominal fluid and serum. P-values below 0.05 were considered significant. Relative abundance between groups was used to estimate the fold-change and to generate volcano plots for group-by-group comparisons. All statistical analyses were performed in R (version 4.2.3) [20]. Significance (–log10 (p value < 0.05), Student´s *t*-test) vs. log2 (mean fold change) were plotted; only significant variables were labeled.

## Results

### Protein profiles in abdominal fluid and serum of CLP and sham-operated animals

Abdominal fluid and serum samples from the peritonitis groups (P1 *n* = 5, P2 *n* = 5, P3 *n* = 5), the peritonitis plus laparoscopic lavage groups (PL1 *n* = 5, PL2 *n* = 5, PL3 *n* = 5), the sham group (S *n* = 5) and the sham plus lavage group (SL *n* = 5) (flowchart shown in Fig. 1) were analyzed for protein content. Two samples of abdominal fluid, both in the sham group, were not analyzed due to inadequate volume.

**Fig. 2:**
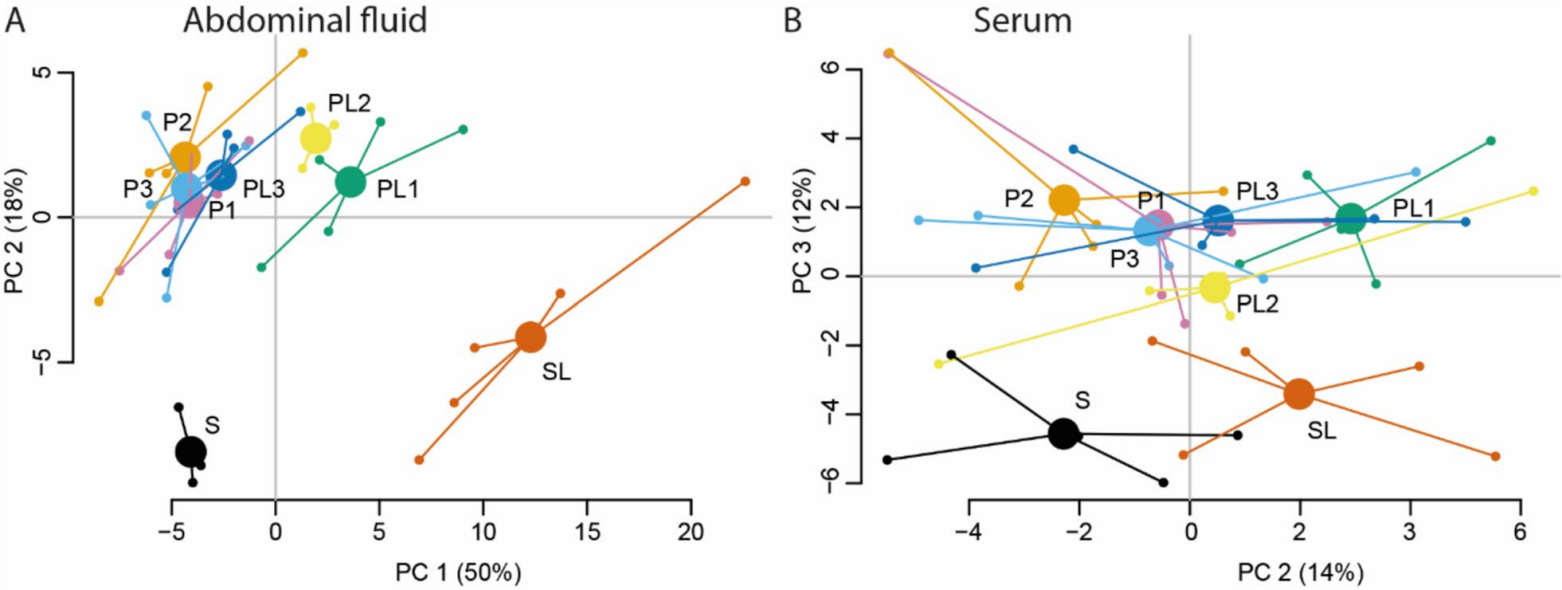
Protein profiles in abdominal fluid and serum. Inflammatory proteins were analyzed by proximity extension immunoassay. Principal component analysis (PCA) including 83 proteins in abdominal fluid (**A**) and 74 proteins in serum (**B**) for peritonitis-afflicted animals (P1, P2 and P3, *n* = 5 per group), peritonitis-afflicted animals treated with laparoscopic lavage (PL1, PL2 and PL3, *n* = 5 per group), sham-operated animals (S, *n* = 3 in abdominal fluid, *n* = 5 in serum) and sham-operated animals treated with laparoscopic lavage (SL, *n* = 5). Lines connect each group to a centroid that shows the combined mean of the group

In abdominal fluid, 91 out of 92 target proteins were successfully quantified, 8 had missing data frequency > 40% and were excluded from further analyses (supplementary Table 1). A principal component analysis (PCA) based on inflammatory proteins (*n* = 83) in abdominal fluid showed that the samples from peritonitis-afflicted animals clustered together and that both the sham group and sham plus lavage group were separated from groups with peritonitis (Fig. 2A). Peritonitis animals treated with laparoscopic lavage and sampled 1 and 2 h after treatment were separated from the untreated peritonitis animals while the laparoscopic lavage group sampled 3 h after lavage was positioned among the untreated peritonitis groups (Fig. 2A).

In serum, 90 out of 92 proteins were successfully quantified, and 16 had missing data frequency > 40% and were not analyzed (supplementary Table 2). A PCA of inflammatory proteins (*n* = 74) in serum illustrated that sham-operated animals and peritonitis-afflicted animals clustered separately (Fig. 2B). Further, a separation was seen between untreated peritonitis animals and the peritonitis animals treated with lavage sampled after 1 h (Fig. 2B).

### Peritonitis induced alterations of protein levels in abdominal fluid and serum

**Fig. 3:**
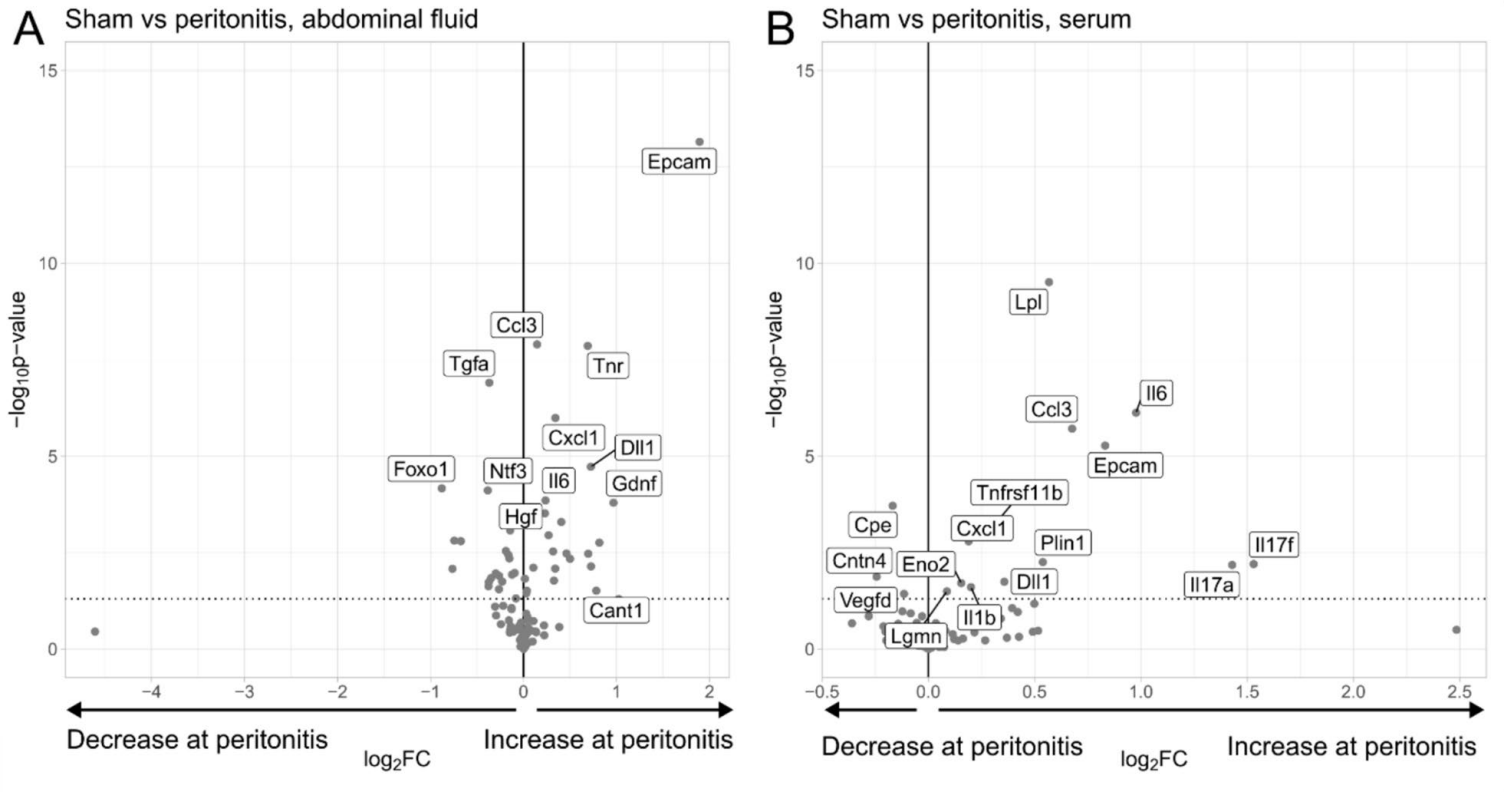
Protein expression in abdominal fluid and serum of peritonitis-afflicted animals vs. sham-operated animals. Inflammatory proteins were analyzed by proximity extension immunoassay. The volcano plots display log2 fold change of the mean protein levels (peritonitis/sham) versus significance (Student’s *t*-test). Some significantly altered proteins (*p* < 0.05) are annotated. Eighty-four proteins were detected in the abdominal fluid of peritonitis-afflicted (*n* = 15) and sham (*n* = 3) animals (**A**), while 71 proteins were identified in serum from peritonitis-afflicted (*n* = 15) and sham (*n* = 5) animals (**B**). However, proteins under the detection level in sham animals but measurable in peritonitis-afflicted animals (CANT1, CCL20 in abdominal fluid; PLIN1, IL17F, IL1B, YES1, ITGB6 in serum) were included

Based on the pattern in the PCA analyses showing tight clustering of the three peritonitis groups; P1, P2 and P3, were analyzed as one group both for abdominal fluid and serum to generate larger sample sizes. In abdominal fluid from the peritonitis-afflicted animals 23 proteins exhibited elevated levels compared to sham (S), while 20 proteins were reduced (Fig. 3A). Notably, proteins such as Epithelial Cell Adhesion Molecule (EPCAM), Tenascin-R (TN-R), Delta-Like Canonical Ligand 1 (DLL1), Glial Cell-Derived Neurotrophic Factor (GDNF), C-X-C Motif Chemokine 9 (CXCL9) demonstrated upregulated expression levels in the peritonitis groups compared to the sham group. Conversely, Forkhead Box Protein O1 (FOXO1), Lipoprotein Lipase (LPL), and Tyrosine-protein Kinase Yes (YES1) were downregulated in peritonitis-afflicted animals when compared to the sham group (Fig. 3A).

In serum, elevated concentrations of 13 proteins, including Lipoprotein Lipase (LPL), Interleukin 6 (IL-6), C–C motif chemokine ligand 3 (CCL3), Epithelial Cell Adhesion Molecule (EPCAM), Interleukin 17A (IL-17A), and Interleukin 17F (IL-17F), were observed in animals with peritonitis when compared to sham subjects while three proteins were found in lower concentrations namely Carboxypeptidase E (CPE), Contactin-4 (CNTN4) and Vascular Endothelial Growth Factor D (VEGF-D) (Fig. 3B).

### Effects of laparoscopic lavage on abdominal inflammatory proteins in peritonitis operated animals

**Fig. 4:**
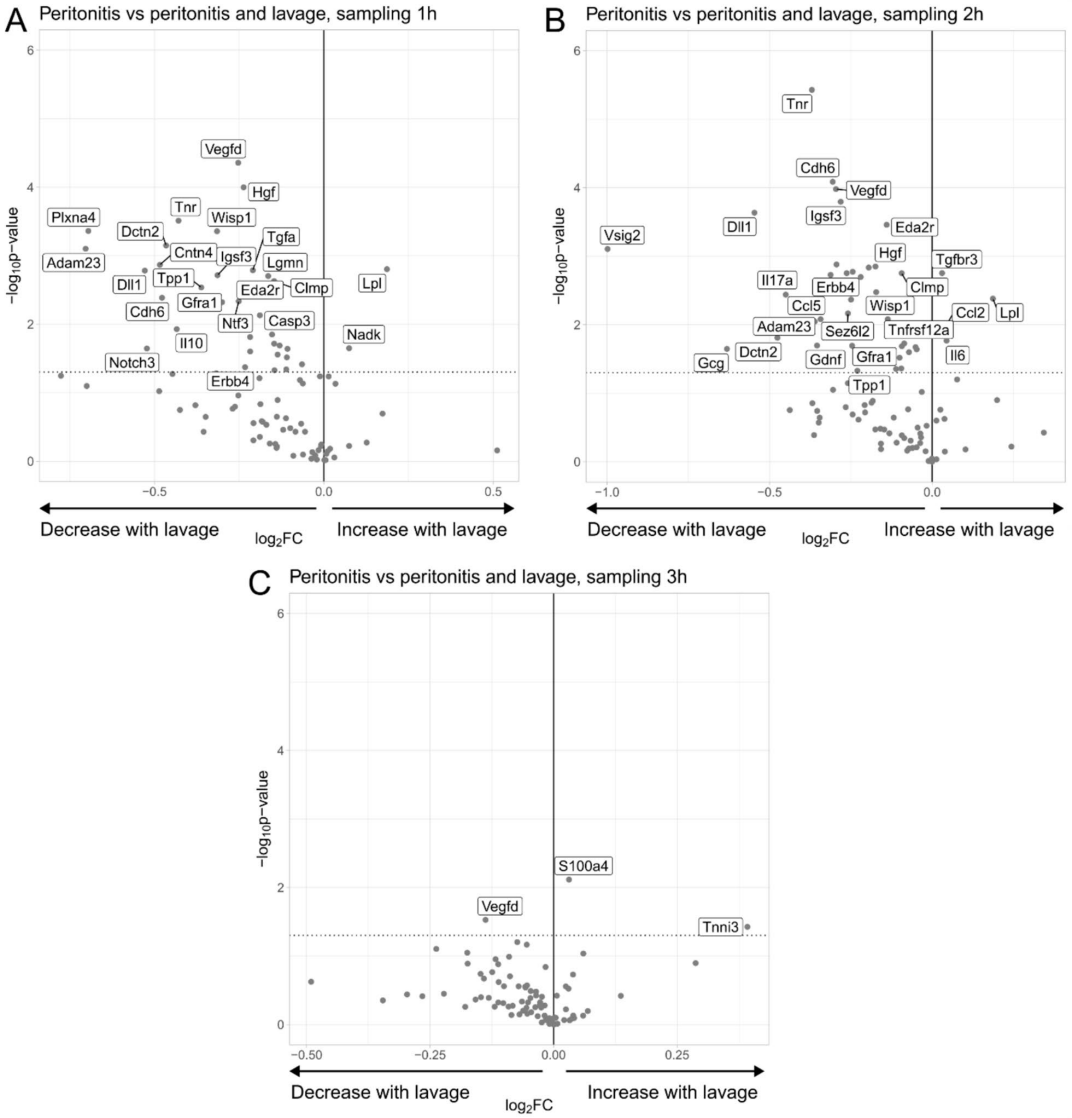
Protein expression in abdominal fluid of peritonitis-afflicted animals treated with laparoscopic lavage vs. peritonitis-afflicted animals. Inflammatory proteins were analyzed by proximity extension immunoassay. Volcano plots illustrating protein expression in abdominal fluid of peritonitis animals (*n* = 5) and peritonitis animals treated with laparoscopic lavage (*n* = 5), sampled one (**A**), two (**B**) or three (**C**) hours after treatment. The volcano plots display log2 fold change of the mean protein levels (peritonitis with laparoscopic lavage/peritonitis) versus significance (Student’s *t*-test). Some significantly altered proteins (*p* < 0.05) are annotated. In the one-hour (**A**), 2 h (**B**), and three-hour (**C**) comparisons, 83, 84, and 84 proteins, respectively, were detected above the threshold in > 40% of samples and included in the data analysis. Notably, in the lavage-treated group, TPP1 and ACVRL1 (**A**), ACVRL1, GCG, and TPP1 (**B**), and CANT1 and ACVRL1 (**C**) exhibited > 40% missing data but were measurable in the peritonitis group, and thus included in the analyses

We evaluated how laparoscopic lavage influenced inflammatory proteins in abdominal fluid in animals with peritonitis at different time points. At one-hour post-treatment, a decline in the concentrations of 33 proteins was observed, while two proteins (LPL and NADK) exhibited elevated concentrations (Fig. 4A). The group sampled 2 h following treatment exhibited a decrease in the concentrations of 35 proteins in animals subjected to laparoscopic lavage, while four proteins (TGFBR3, LPL, CCL2 and IL6) were found in elevated concentrations compared to time-matched peritonitis controls (Fig. 4B). The 3 h post-treatment group showed a decrease in the concentration of one protein in laparoscopic lavage-treated animals (VEGF-D) and two proteins (S100A4 and TNNI3) exhibited elevated concentrations as compared to peritonitis controls (Fig. 4C).

The results from the laparoscopic lavage-treated groups, at both one- and two-hours post-treatment, revealed significant alterations in numerous proteins. Among these, 27 proteins were consistently affected at both time points when compared to the peritonitis groups. Proteins such as TNR, Cadherin 6 (CDH6), DLL1, VEGF-D, disintegrin and metalloproteinase domain-containing protein 23 (ADAM23) were found in lower levels in both comparisons. Conversely, elevated levels of lipoprotein lipase (LPL) were observed.

### Effects of laparoscopic lavage on abdominal inflammatory proteins in sham-operated animals

**Fig. 5:**
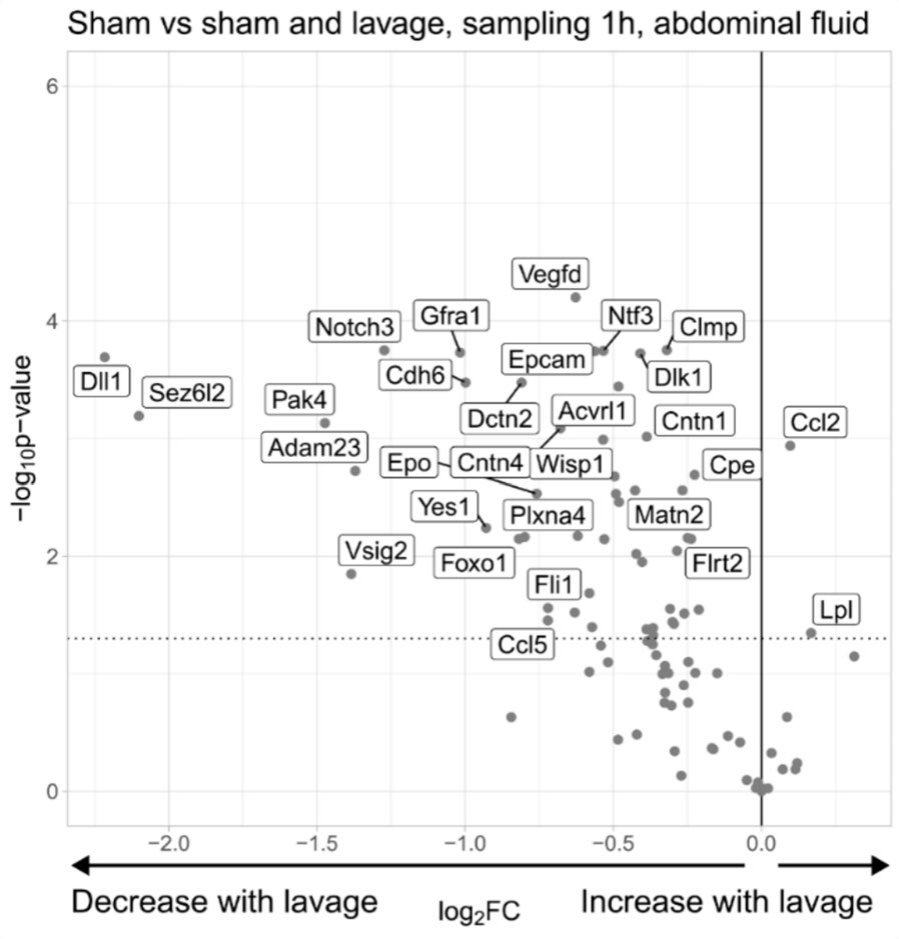
Protein expression in abdominal fluid of sham animals vs. sham animals treated with laparoscopic lavage. Inflammatory proteins were analyzed by proximity extension immunoassay in abdominal fluid of sham animals (*n* = 3) and sham animals treated with laparoscopic lavage (*n* = 5), sampled one-hour post-treatment. The volcano plot displays log2 fold change of the mean protein levels (sham with laparoscopic lavage/sham) versus significance (Student’s *t*-test). Eighty-four proteins were detected above the threshold in > 40% of samples and included in the data analysis. Eight proteins had > 40% missing data in the lavage-treated animals but measurable in sham-operated animals and were included in the data analysis (ACVRL1, EPO, ITGB6, TPP1, VSIG2, IL10, GHRL and IL1A)

When analyzing the outcomes of the sham (S) animals compared to sham animals subjected to laparoscopic lavage (SL), we observed significant alterations in 52 out of 83 analyzed proteins in abdominal fluid. Fifty proteins, including VEGF-D, DLL1, CDH6, ADAM23, TNR, IL-17A, IL-17F, and VSIG2, exhibited lower concentrations in animals treated with lavage. Conversely, increased levels of Chemokine (C–C motif) Ligand 2 (CCL2) and LPL were detected following lavage (Fig. 5).

### Effects on inflammatory proteins in serum after laparoscopic lavage

In serum, no consistent pattern of protein alterations was observed when comparing the laparoscopic lavage-treated groups with peritonitis-afflicted animals across the different time points (supplementary Fig. 1 A, B and C). The sham-sham lavage comparison exhibited significantly lower levels of several proteins such as VEGF-D, Carboxypeptidase E (CPE), APBB1IP, Contactin 4 (CNTN4) in lavage-treated animals compared to sham (supplementary Fig. 2).

## Discussion

In this study, we characterized the inflammatory reaction in rats affected by CLP-induced peritonitis, showing elevated levels of interleukins, chemokines, and cell adhesion molecules in both abdominal fluid and serum. We also found that the reduction in inflammatory proteins in the abdominal fluid after laparoscopic lavage was mainly seen one and two hours after treatment indicating a time dependent effect.

The study suggests that our model for inducing peritonitis in rats is safe, robust, and reproducible. The peritonitis-afflicted animals showed raised levels of several proinflammatory proteins indicating an acute inflammation such as cell adhesion molecules EPCAM and TN-R, the chemokine CCL3 and several well-known proinflammatory cytokines such as IL-17A, IL-1A and TNF in abdominal fluid, compared to sham. These results were consistent with our previous work [16].

It remains uncertain whether the observed effect of the lavage treatment in abdominal fluid is a result of dilution due to the extensive use of saline or if other mechanisms are at play. One proposed hypothesis is that during laparoscopic lavage saline reacts with carbon dioxide, resulting in a more acidic environment which could have a direct effect on the inflammatory response. Previous studies have shown that peritoneal acidosis suppressed the immune system by increasing serum IL10 and decreasing serum TNFα levels in rats with lipopolysaccharide (LPS)-induced sepsis [21, 22].

We found that laparoscopic lavage leads to a reduction in the levels of various proinflammatory proteins present in the abdominal fluid, including the growth factor VEGF-D, cell adhesion proteins TN-R and CDH6, as well as DLL1, which plays a crucial role in cell-to-cell communication. These results suggest that laparoscopic lavage may have a selective and targeted anti-inflammatory effect, particularly on certain proinflammatory proteins within the abdominal fluid. The consistent reduction in VEGF-D levels across all groups, including both peritonitis-afflicted and sham-operated animals, indicates that laparoscopic lavage may specifically downregulate pathways associated with vascular growth and inflammation. This observation is significant because VEGF-D is not only involved in inflammation, but also in angiogenesis, implying that laparoscopic lavage might influence processes related to tissue repair and regeneration [23, 24]. In contrast, lipoprotein lipase (LPL) was found in elevated levels in abdominal fluid after laparoscopic lavage both in CLP operated animals and sham-operated controls possibly indicating a direct influence of the lavage treatment. Lipoprotein lipase is well known as an endothelial surface enzyme that regulates triglycerides in the bloodstream. The limited number of studies investigating LPL in the context of inflammation have primarily focused on cardiovascular and neurological diseases, where LPL exhibits proinflammatory effects by modulating immune cell responses and macrophage activity in the arterial wall [25, 26]. However, its role in acute abdominal inflammation remains unclear, particularly regarding the observed increase in LPL levels following laparoscopic lavage. One plausible hypothesis is that laparoscopic lavage amplifies the peritoneal recruitment of macrophages through raised levels of LPL, thereby modulating the local immune response against infection.

In our study the effects of laparoscopic lavage on protein expression patterns are similar in abdominal fluid and serum (Fig. 2A, B). However, in serum there is no consistent alteration of protein patterns when comparing different timepoints and the results are thereby difficult to interpret. This may be due to a delay of the systemic inflammatory response in comparison to the immediate local response in the abdominal cavity and our sampling time points may have been too early to capture changes in serum.

In our experimental model the ischemic cecum was left in the abdomen which would sustain the inflammatory response after the lavage treatment. This could explain why, in our model, the effect of lavage seemed to be reduced with time. In humans with purulent peritonitis due to perforated diverticulitis there is no ischemic tissue in the abdomen, so in that sense the model does not perfectly mimic the clinical situation. One could consider improving the model by performing a second laparotomy 8 h after CLP and remove the ischemic tissue, but this could alter the intra-abdominal conditions and would certainly inflict more trauma to the animal which could influence the inflammatory response. Another approach could be to do a “real” laparoscopy and surgically remove the ischemic cecum during the laparoscopic intervention [27]. However, it is much more time consuming and technically challenging, still it might come to use in future experiments.

This model was developed to simulate the clinical scenario of perforated diverticulitis with purulent peritonitis and treatment with laparoscopic lavage. During this procedure, carbon dioxide is insufflated into the abdomen, traditionally at a pressure of 12–15 mmHg. However, recent trends favor reducing this pressure to minimize potential adverse effects. A 2023 meta-analysis reported that intra-abdominal pressures below 10 mmHg were associated with fewer mild complications, reduced postoperative pain and nausea, and a shorter length of hospital stay [28]. In rats weighing 250–375 g, a pressure of 8 mmHg was used, which is expected to adequately simulate the clinical setting and enable meaningful interpretation of the outcomes within this model.

This study has several limitations. It is an explorative study with small group sizes and the sham group comprised only three evaluable samples from the abdominal fluid. The choice of analysis method can also be seen as a limitation, as we did not analyze all present proteins, but rather a preselected set. Some proteins were under the limit of detection which could be due to a deficiency in the analysis method or an actual low protein-level. Furthermore, correction for multiple testing was not used in the statistical analysis, which most likely has led to some false positive results. In this relatively small exploratory and hypothesis-generating study we chose not to correct for this possible error and to use *p* < 0.05 as cut of limit for significant results.

This exploratory study aimed to generate hypotheses rather than test predefined ones. Therefore, no sample size calculation was performed during the study design. However, future studies building on these findings and testing specific hypotheses will include sample size calculations.

## Conclusion

This study demonstrates that our previously described model for purulent peritonitis in rats is reproducible, with inflammatory proteins serving as reliable markers for this condition. Laparoscopic lavage significantly reduced the levels of several inflammatory proteins in abdominal fluid, with the most pronounced effects observed in samples collected one to two hours post-treatment. This experimental model of purulent peritonitis offers a platform for future studies aimed to clarify the underlying mechanisms of action of laparoscopic lavage.

## Supplementary Information

Supplementary Material 1: Figure 1 Protein expression in serum of peritonitis-afflicted animals treated with laparoscopic lavage vs. peritonitis-afflicted animals. Inflammatory proteins were analyzed by proximity extension immunoassay. Volcano plots illustrating protein expression in serum of peritonitis animalsand peritonitis animals treated with laparoscopic lavage, sampled one, twoor threehours after treatment. The Volcano plots display log2 fold change of the mean protein levelsversus significance. Significantly altered proteinsare annotated. In the one-hour comparison, 68 out of 92 proteins were detected above the threshold in >40% of samples and included in the analysis. In the two-hourand three-hourcomparisons, 73 proteins met this criterion. Among these, VSIG2, ITGB6, and PAK4, as well as CCL20, had >40% missing data in the lavage-treated group but were measurable in the peritonitis group, and were thus included in the analysis.

Supplementary Material 2: Figure 2 Protein expression in serum of sham animals vs. sham animals treated with laparoscopic lavage. Inflammatory proteins were analyzed by proximity extension immunoassay in serum of sham animalsand sham animals treated with laparoscopic lavage, sampled one-hour post-treatment. The Volcano plot displays log2 fold change of the mean protein levelsversus significance. Sixty-nine proteins were detected above the threshold in >40% of samples. Among these, four proteinsexhibited >40% missing data in lavage-treated animals but were measurable in sham-operated animals and were therefore included in the data analysis.

Supplementary Material 3: Table 1, proteins with missing data frequency >40% in abdominal fluid. Table 2, proteins with missing data frequency >40% in serum.

## Data Availability

The datasets used and analyzed during the current study are available from the corresponding author on reasonable request.
