# Supplementary material for: Laparoscopic lavage in a purulent peritonitis model: impact on inflammatory proteins
Source: Eur J Med Res. 2025 Mar 18;30:180. doi: 10.1186/s40001-025-02445-2 (PMC11917159; doi:10.1186/s40001-025-02445-2)
Supplement: Supplementary file 1 — Supplementary Material 1: Figure 1 Protein expression in serum of peritonitis-afflicted animals treated with laparoscopic lavage vs. peritonitis-afflicted animals. Inflammatory proteins were analyzed by proximity extension immunoassay. Volcano plots illustrating protein expression in serum of peritonitis animalsand peritonitis animals treated with laparoscopic lavage, sampled one, twoor threehours after treatment. The Volcano plots display log2 fold change of the mean protein levelsversus significance. Significantly altered proteinsare annotated. In the one-hour comparison, 68 out of 92 proteins were detected above the threshold in >40% of samples and included in the analysis. In the two-hourand three-hourcomparisons, 73 proteins met this criterion. Among these, VSIG2, ITGB6, and PAK4, as well as CCL20, had >40% missing data in the lavage-treated group but were measurable in the peritonitis group, and were thus included in the analysis. [file 40001_2025_2445_MOESM1_ESM.docx]

**Supplementary figure 1**


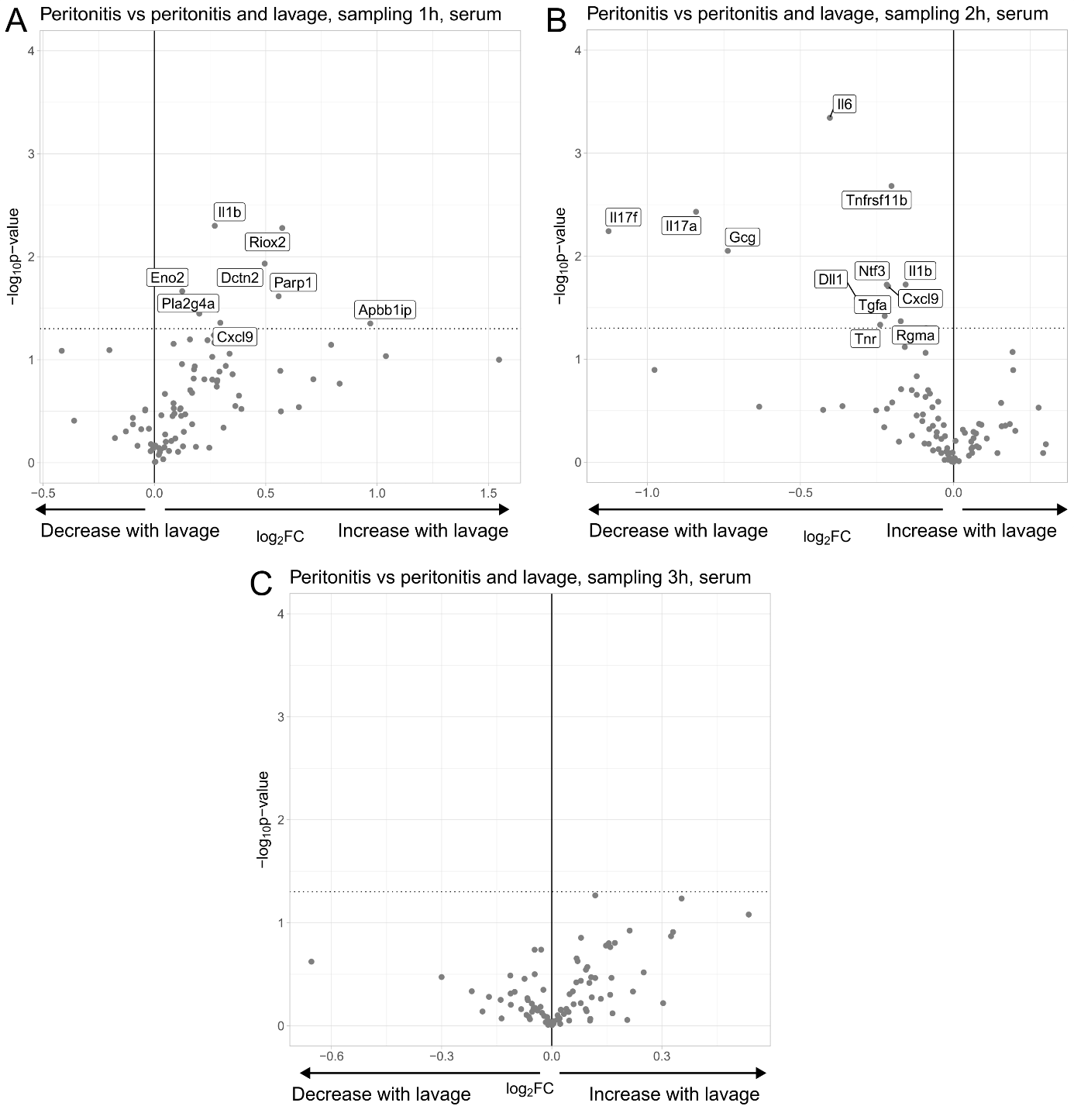


**Supplementary figure 1** - **Protein expression in serum of peritonitis-afflicted animals treated with laparoscopic lavage vs. peritonitis-afflicted animals.** Inflammatory proteins were analyzed by proximity extension immunoassay. Volcano plots illustrating protein expression in serum of peritonitis animals (n=5) and peritonitis animals treated with laparoscopic lavage (n=5), sampled one (A), two (B) or three (C) hours after treatment. The Volcano plots display log2 fold change of the mean protein levels (peritonitis with laparoscopic lavage/peritonitis) versus significance (Student’s t-test). Significantly altered proteins (p<0.05) are annotated.

In the one-hour comparison (A), 68 out of 92 proteins were detected above the threshold in >40% of samples and included in the analysis. In the two-hour (B) and three-hour (C) comparisons, 73 proteins met this criterion. Among these, VSIG2, ITGB6, and PAK4 (B), as well as CCL20 (C), had >40% missing data in the lavage-treated group but were measurable in the peritonitis group, and were thus included in the analysis.
