# Supplementary material for: Laparoscopic lavage in a purulent peritonitis model: impact on inflammatory proteins
Source: Eur J Med Res. 2025 Mar 18;30:180. doi: 10.1186/s40001-025-02445-2 (PMC11917159; doi:10.1186/s40001-025-02445-2)
Supplement: Supplementary file 2 — Supplementary Material 2: Figure 2 Protein expression in serum of sham animals vs. sham animals treated with laparoscopic lavage. Inflammatory proteins were analyzed by proximity extension immunoassay in serum of sham animalsand sham animals treated with laparoscopic lavage, sampled one-hour post-treatment. The Volcano plot displays log2 fold change of the mean protein levelsversus significance. Sixty-nine proteins were detected above the threshold in >40% of samples. Among these, four proteinsexhibited >40% missing data in lavage-treated animals but were measurable in sham-operated animals and were therefore included in the data analysis. [file 40001_2025_2445_MOESM2_ESM.docx]

**Supplementary figure 2**


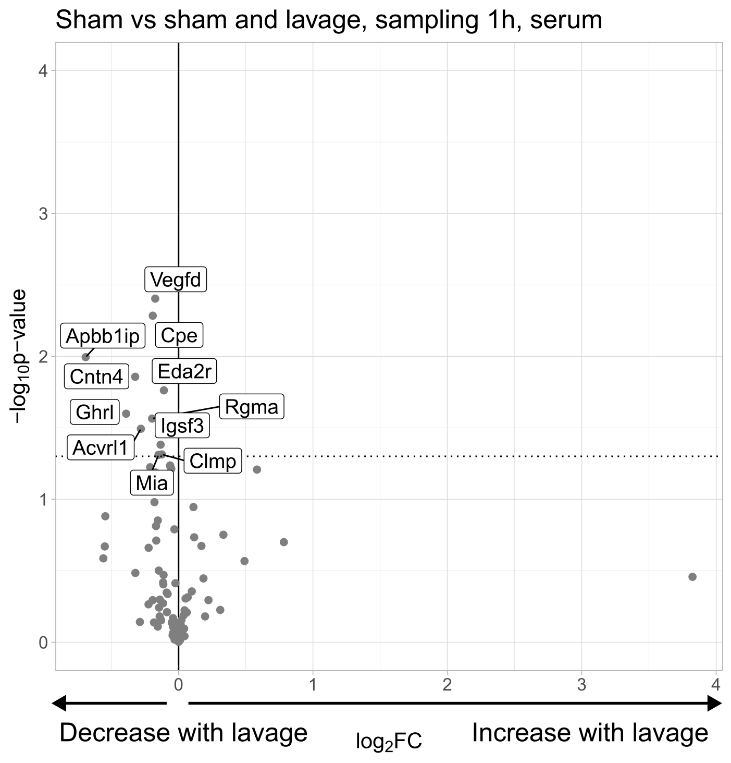


**Supplementary figure 2 - Protein expression in serum of sham animals vs. sham animals treated with laparoscopic lavage.** Inflammatory proteins were analyzed by proximity extension immunoassay in serum of sham animals (n=5) and sham animals treated with laparoscopic lavage (n=5), sampled one-hour post-treatment. The Volcano plot displays log2 fold change of the mean protein levels (sham with laparoscopic lavage/sham) versus significance (Student’s t-test). Sixty-nine proteins were detected above the threshold in >40% of samples. Among these, four proteins (APBB1IP, ACVRL1, VSIG2, and GCG) exhibited >40% missing data in lavage-treated animals but were measurable in sham-operated animals and were therefore included in the data analysis.
