# Supplementary material for: Laparoscopic lavage in a purulent peritonitis model: impact on inflammatory proteins
Source: Eur J Med Res. 2025 Mar 18;30:180. doi: 10.1186/s40001-025-02445-2 (PMC11917159; doi:10.1186/s40001-025-02445-2)
Supplement: Supplementary file 3 — Supplementary Material 3: Table 1, proteins with missing data frequency >40% in abdominal fluid. Table 2, proteins with missing data frequency >40% in serum. [file 40001_2025_2445_MOESM3_ESM.docx]

**Supplementary tables**

Supplementary table 1, proteins with missing data frequency >40% in abdominal fluid (n=9)

| Protein name | Missing data % | LOD* |
| --- | --- | --- |
| GCG | $74\%$ | $1,47$ |
| CLSTN2 | $95\%$ | $2,22$ |
| CANT1 | $52\%$ | $0,56$ |
| AHR | $69\%$ | $2,01$ |
| FAS | $100\%$ | $1,39$ |
| IL23R | $98\%$ | $0,53$ |
| CSF2 | $62\%$ | $1,13$ |
| GHRL | $45\%$ | $2,27$ |
| IL5 | $98\%$ | $2,97$ |

**Limit of detection*

Supplementary table 2, proteins with missing data frequency >40% in serum (n=18)

| Protein name | Missing data % | LOD* |
| --- | --- | --- |
| GDNF | $52\%$ | $1,87$ |
| FOXO1 | $64\%$ | $1,82$ |
| CLSTN2 | $95\%$ | $2,10$ |
| CA13 | $57\%$ | $1,11$ |
| CANT1 | $64\%$ | $1,08$ |
| PPP1R2 | $71\%$ | $1,98$ |
| AHR | $100\%$ | $2,07$ |
| FAS | $100\%$ | $1,57$ |
| IL1A | $45\%$ | $2,22$ |
| IL23R | 95% | 0,35 |
| IL10 | 67% | 1,61 |
| ACVRL1 | 55% | 2,18 |
| CSF2 | 95% | 1,34 |
| ITGB1BP2 | 48% | 0,94 |
| IL5 | 98% | 2,20 |
| CCL20 | 55% | 1,96 |
| FLI1 | 83% | 2,79 |
| TNF | 64% | 0,50 |

**Limit of detection*
